# Investigating the Impact of COVID-19 Infection on Dry Eye Parameters

**DOI:** 10.3390/diagnostics13091524

**Published:** 2023-04-24

**Authors:** Xulin Liao, Arthur Chun Chi Wong, June Oi Yau Wong, Ruofan Jia, Wanxue Chen, Hanson Yiu Man Wong, Fatema Mohamed Ali Abdulla Aljufairi, Kenneth Ka Hei Lai, Zhichao Hu, Yingying Wei, Clement Chee Yung Tham, Chi Pui Pang, Kelvin Kam Lung Chong

**Affiliations:** 1 Department of Ophthalmology and Visual Sciences, The Chinese University of Hong Kong, Hong Kong SAR, China; liaoxulin@link.cuhk.edu.hk (X.L.); wanxue.chen@link.cuhk.edu.hk (W.C.);; 2 Department of Statistics, The Chinese University of Hong Kong, Hong Kong SAR, China; 3 Department of Ophthalmology, Salmaniya Medical Complex, Government Hospitals, Manama 435, Bahrain; 4 Department of Ophthalmology, Tung Wah Eastern Hospital, Hong Kong SAR, China

**Keywords:** dry eye, COVID-19, lipid layer thickness, meibomian gland dysfunction, non-invasive keratography tear break-up time

## Abstract

Purpose: This study aims to compare dry eye parameters before and after COVID-19 infection in dry eye patients. Methods: We included 44 dry eye patients (88 eyes) from our existing dry eye cohort, with 22 belonging to the post-COVID-19 group due to a prior COVID-19 infection and the other 22 forming the non-COVID-19 group as they had no history of COVID-19. We examined and compared the dry eye parameters of the post-COVID-19 group, including the ocular surface disease index (OSDI), Schirmer’s test results (ST), non-invasive Keratography tear break-up time (NIKBUT), lipid layer thickness (LLT), Meibomian gland dysfunction (MGD), and the grading of papillae and follicles, both before and after the COVID-19 infection. We also compared the dry eye parameters difference of the post-COVID-19 group with the non-COVID-19 group. Results: The post-COVID-19 group was comprised of individuals with an average age of 38.36 ± 14.99 years, of which 82% were female. The time interval between the two tests was 16.92 ± 5.40 months, which did not differ significantly from the non-COVID-19 group. Compared to the pre-COVID-19 eyes, the post-COVID-19 eyes showed a significant decrease in the average LLT (52.86 ± 18.00 nm vs. 63.00 ± 22.40 nm, *p* < 0.001), as well as the maximum LLT (67.89 ± 20.81 nm vs. 78.48 ± 20.55 nm, *p* < 0.001). The MGD in both the upper (1.75 ± 0.84) and lower eyelids (1.43 ± 0.73) worsened after a COVID-19 infection. Additionally, the grading of papillae was worse following a COVID-19 infection (0.61 ± 0.69 vs. 0.16 ± 0.37, *p* < 0.001). The multivariate linear regression model revealed a negative association between COVID-19 infection and NIKBUT-average (β = −2.98, 95%CI: (−5.82, −0.15), *p* = 0.039), LLT-average (β = −14.12, 95%CI: (−22.66, −5.59), *p* = 0.001), and LLT max (β = −15.65, 95%CI: (−23.09, −8.20), *p* < 0.001). Conclusion: From preliminary results, we concluded that dry eye patients who have been infected with COVID-19 appear to have a more severe dry eye condition, as evidenced by lower LLT, worse papillae and MGD, and shorter NIKBUT. It is important to raise awareness of this potential long-term symptom of COVID-19, especially among existing dry eye patients.

## 1. Introduction

Dry eye syndrome is a prevalent ocular condition that affects millions of people globally. Studies have found that, in the United States, more than 16 million (6.8%) of the population experience dry eye symptoms [1]. Among contact lens wearers in the United Kingdom, the prevalence of dry eye was found to be approximately 50% [2], while among Chinese high school students in China, the prevalence was approximately 70.5% [3]. The 2017 Tear Film and Ocular Surface Society Dry Eye Workshop (TFOS DEWS II) [4] categorized dry eye into two main types: evaporative and aqueous deficient dry eye. On the other hand, the Asia Dry Eye Society (ADES) included an additional subtype of tear-deficient dry eye called mucin-deficient dry eye [5]. Dry eye has been associated with a number of risk factors, including advancing age, female gender, autoimmune diseases, diabetes, thyroid disorders, exposure to dry environments, use of contact lenses, prolonged periods of screen time, and other factors [6,7,8]. According to our previous study, there is an association between the viral loads of COVID-19 and evaporative dry eye among COVID-19 patients who were discharged from the hospital after one to three months [9]. 

COVID-19, caused by the novel coronavirus SARS-CoV-2, is an infectious disease first identified in humans in December 2019 and has since become a global pandemic [10,11]. The World Health Organization has reported that as of February 2023, there have been an estimated six million deaths and over 700 million confirmed cases of COVID-19 worldwide. The Hong Kong government has reported over 2.88 million confirmed cases and over 10,000 deaths in Hong Kong alone. Among the various strains of SARS-CoV-2, including Alpha, Beta, Gamma, Delta, and Omicron, each strain has unique characteristics in terms of transmissibility and pathogenicity [12]. Early evidence suggests that Omicron may cause milder symptoms and be associated with fewer hospitalizations and deaths than previous variants [13]. 

Despite the ongoing efforts to manage the COVID-19 pandemic, many individuals are still experiencing persistent symptoms of the disease. Long COVID [14], also referred to as post-acute sequelae of SARS-CoV-2 infection, is a condition in which individuals continue to experience symptoms of COVID-19 for weeks or even months after the initial acute phase of the illness has passed. These symptoms can vary greatly and may include fatigue, shortness of breath, chest pain, joint pain, cognitive difficulties, sleep disturbances, and depression [15]. Additionally, those affected by long COVID may experience damage to multiple organs, including the lungs, heart, and brain, which can result in long-term disability [16]. Building on our prior research [9], we sought to investigate the potential long-term effects of the novel coronavirus on eye health, particularly with regard to the development of dry eye.

## 2. Methods

### 2.1. Study Subject

**Figure 1:**
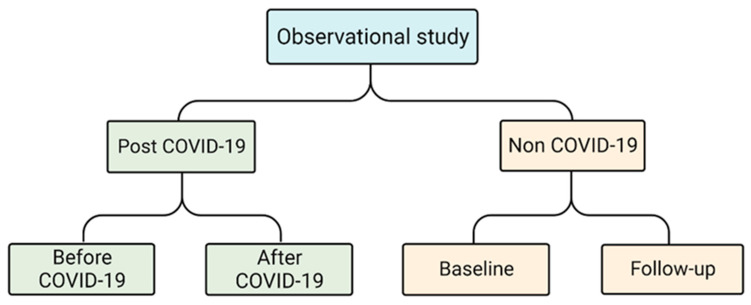
Flow chart of the study design.

This was a longitudinal, observational study. Our dry eye cohort, which was conducted between June 2021 and June 2022, includes 358 patients who had been diagnosed with dry eye disease at the Chinese University of Hong Kong Medical Centre or the Chinese University of Hong Kong Eye Centre. From our dry eye cohort, we included patients with an Ocular Surface Disease Index (OSDI) [17] score of 13 or above and available baseline data indicating they had previously been infected with COVID-19. A non-COVID-19 group was also included, consisting of patients with an OSDI score of 13 or above and available baseline data indicating they had not been infected with COVID-19. Both groups were invited to attend a follow-up test (Figure 1). The study excluded patients with glaucoma, thyroid-eye disease, tumors, conjunctivitis, or other eye diseases, as well as some who had undergone radiotherapy. COVID-19 diagnosis was made using conjunctival and nasopharyngeal RT-PCR tests, rapid antigen tests, or a combination of both, as required by the HKSAR government.

### 2.2. Ophthalmic Examinations

Comprehensive ophthalmic examinations were administered to all subjects to evaluate the dry eye parameters, which included measuring the corrected distance visual acuity and assessing the ocular surface. The assessment covered aqueous parameters, lipid parameters, and conjunctiva. The Schirmer’s test, slit-lamp examination, and the Oculus^®^ Keratograph 5M and LipiView^®^ II Ocular Surface Interferometer were used in the examination process.

During the Schirmer I test, a Schirmer’s test strip was placed in the lateral third of the lower fornix of the patient’s eye for five minutes while the patient was instructed to keep their eyes closed without any anesthesia given. The length of the moistened area on the strip was then recorded in millimeters per 5 min. If the length of wetting is below 10 mm, it may indicate that the patient has dry eye syndrome or another condition that affects tear production [18].

The grading system proposed by Fukushima et al. was utilized in the slit-lamp examination to assess conjunctival follicles and papillae. A four-point scale was used for grading, where Grade 3 indicated the presence of 20 or more follicles for follicles and a papillae size of 0.6 mm or more for papillae. Grade 2 indicated 10–19 follicles for follicles and a papillae size of 0.3–0.5 mm for papillae. Grade 1 indicated the presence of 1–9 follicles for follicles and a papillae size of 0.1–0.2 mm for papillae. Grade 0 indicated the absence of both follicles and papillae [19].

The LipiView^®^ II Ocular Surface Interferometer [20] was used to evaluate the lipid layer thickness (LLT) and meibomian gland morphology for each subject. The LLT was measured by projecting a flashing light over the lower one-third of the cornea for 20 s while natural blinking occurred. The LipiView^®^ defined an upper cut-off of 100 nm. The average, maximum, and minimum LLT were recorded. Meibomian gland (MG) morphology was assessed by everting the lower and upper eyelids and obtaining LipiView^®^ II images of the lower eyelid using a penetrating infrared light source. The meiboscore was assigned based on the following scale: grade 0 = 0% loss of MG; grade 1 < 33% loss of the total MG area; grade 2 is between 33% and 67% loss of the total MG area; and grade 3 > 67% loss of the total MG area [21].

The Objective Oculus^®^ Keratograph 5M was used to measure tear meniscus height (TMH) and non-invasive Keratography tear break-up time (NIKBUT). A greyscale image of the eye was captured, and TMH was measured manually using a cursor in the system. NIKBUT was measured as first NIKBUT and average NIKBUT in seconds, which were detected automatically. The first NIKBUT was the measurement of the time between the last complete blink and the first disruption of projected rings on the cornea. Patients were asked to avoid blinking during this test [22].

### 2.3. Statistical Analyses

The data analysis was conducted using IBM SPSS 23.0 (IBM SPSS Inc., Armonk, NY, USA, IBM Corp) and R (The R Project for Statistical Computing, version 4.2.1). Continuous variables were presented as means ± standard deviations, while categorical variables were presented as percentages. In Table 1, Fisher’s exact test was used for categorical variables, and Student’s *t*-test was used for continuous variables to compare differences between the two groups. In Table 2, the paired *t*-test was used for continuous variables to compare the same eye before and after infection. In Table 3, the Student’s *t*-test was used to compare the difference in dry eye parameters between groups. In Table 4, univariate and multivariate linear regression analyses were performed to investigate the association between COVID-19 infection and dry eye parameters in dry eye patients. The multivariable regression model adjusts for age and sex, and the generalized estimating equation was used to account for inter-eye correlation. Results were considered statistically significant if the *p*-value was less than 0.05.

## 3. Results

This study included 44 dry eye patients with 88 eyes in total. The post-COVID-19 group consisted of 22 patients and 44 eyes, while the non-COVID-19 group also had 22 patients and 44 eyes. The mean age of the post-COVID group was 38.36 ± 14.99 years old, and 18 were female. The time interval between the first and second tests was 16.92 ± 5.40 months, and between the COVID-19 infection and the second test was 4.61 ± 2.39 months. There were no significant differences in these data compared to the non-COVID-19 group. However, the vaccination rate was significantly lower in the post-COVID-19 group compared to the non-COVID-19 group (*p* = 0.001) (Table 1). 

**Figure 2:**
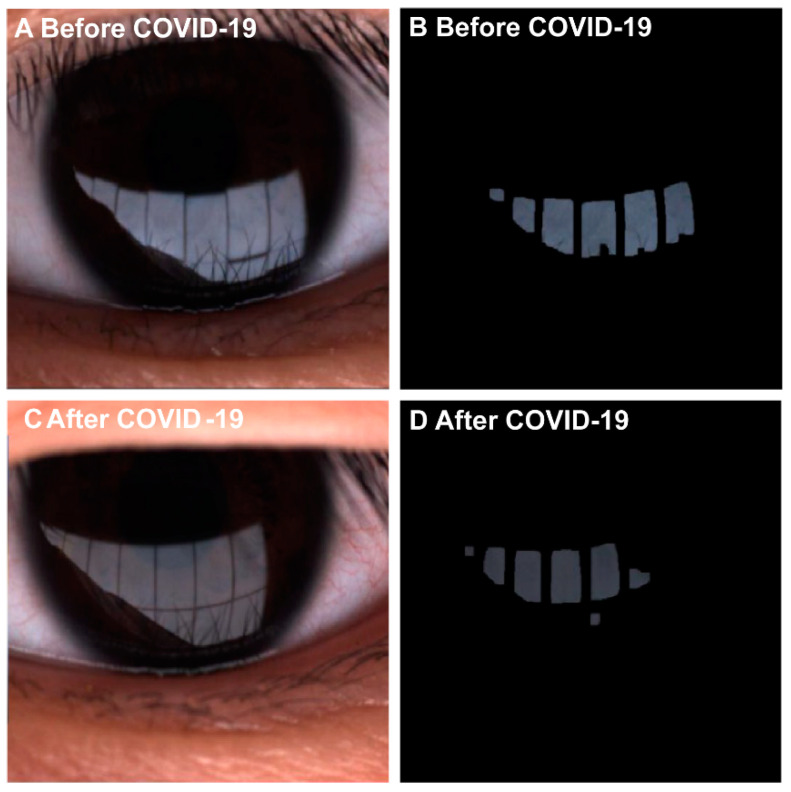
Dry eye patient lipid image report from LipiView II Ocular Surface Interferometer. (**A**,**B**) Lipid image of dry eye patient before COVID-19 infection. (**C**,**D**) Lipid image of dry eye patient after COVID-19 infection.

Table 2 compared the changes in dry eye parameters for both post-COVID-19 and non-COVID-19 dry eye patients. In terms of lipid parameters, the post-COVID-19 group showed a significant decrease in LLT-average, from 63.00 ± 22.40 nm to 52.86 ± 18.00 nm (*p* < 0.001), and in LLT-max, from 78.48 ± 20.55 nm to 67.89 ± 20.81 nm (*p* < 0.001) (Figure 2). Meiboscore in the upper eyelid worsened from 1.55 ± 0.73 to 1.75 ± 0.84 (*p* = 0.011), and the lower eyelid gland also worsened from 1.30 ± 0.51 to 1.43 ± 0.73 (*p* = 0.001). Papillae increased from 0.16 ± 0.37 to 0.61 ± 0.69 (*p* = 0.001). However, there were no significant differences in other parameters, including visual acuity, OSDI, NIKBUT, aqueous parameters, and follicles. In contrast, the non-COVID-19 group did not show significant changes in the above parameters.

Table 3 presents a comparison of the difference in dry eye parameters between the follow-up time and the baseline for the post-COVID-19 patients and non-COVID-19 dry eye patients. The difference in values for the post-COVID-19 group was calculated by subtracting the values after COVID-19 from those before COVID-19, while for the non-COVID-19 group, the difference was calculated by subtracting the follow-up values from the baseline values. A negative value indicates a decrease, while a positive value indicates an increase. The NIKBUT-average difference in the non-COVID-19 group was 1.69 ± 5.78 s, while in the post-COVID-19 group it was −1.30 ± 6.11 s (*p* = 0.02). The LLT-average difference in the non-COVID-19 group was 5.16 ± 19.73 nm, while in the post-COVID-19 group it was −10.14 ± 16.29 nm (*p* < 0.001). The LLT-max difference in the non-COVID-19 group was 5.11 ± 18.72 nm, while in the post-COVID-19 group it was −10.59 ± 15.48 nm (*p* < 0.001). There were no significant differences in the other parameters. In summary, the comparison of the differences between the two groups showed that post-COVID-19 patients had a shorter tear film breakup time and a thinner lipid layer thickness.

Table 4 displays the association between COVID-19 infection and differences in dry eye parameters among patients with dry eye. We used linear regression to investigate the relationship between COVID-19 and all differences in dry eye parameters, with the differences in dry eye parameters as the dependent variables. In the multivariate regression model, we adjusted for age and sex and found that the NIKBUT-average (β = −2.98, 95%CI: (−5.82, −0.15), *p* = 0.039), LLT-average (β = −14.12, 95%CI: (−22.66, −5.59), *p* = 0.001), and LLT max (β = −15.65, 95%CI: (−23.09, −8.20), *p* < 0.001) were negatively associated with COVID-19 infection.

## 4. Discussion

Our study focuses on the dry eye condition of patients with primary dry eye syndrome six months after contracting COVID-19. It is the first study to compare detailed dry eye examination data of the same patients before and after contracting COVID-19. These patients exhibited a decline in meibomian gland function, reduced lipid layer thickness, increased conjunctival follicles, and shorter tear film break-up time. Specifically, the difference in average thickness of the lipid layer was observed to decrease by approximately 14 nm compared to patients who did not contract COVID-19.

Our study found that the atrophy of the meibomian glands worsened, with a more pronounced thinning of the lipid layer. According to some studies [23,24], the SARS-CoV-2 virus can bind to the ACE2 receptors on host cells, causing gland dysfunction. As the ACE2 receptor is present in various cells, including those in the lung, heart, kidney, gastrointestinal tract, and meibomian glands [25], our hypothesis is that the virus has a direct impact on gland function. Some studies support this hypothesis [9,26], suggesting that COVID-19 may reduce the function of the meibomian glands. However, other studies propose that dry eye symptoms may be due to the use of face masks or changes in temperature that cause rapid evaporation of the tear film on the ocular surface [27]. COVID-19 can lead to inflammation in different organs of the body, such as the lungs, heart, brain, and others. This inflammation can be caused by an overactive immune response triggered by the virus, leading to tissue damage and other complications. Therefore, we speculate that inflammation in the conjunctiva [28], which is easily observable as the eye appears red, may occur after contracting COVID-19. However, dysfunction of the meibomian glands is not easily noticeable and may be more persistent in nature.

It has been suggested that the altered ocular surface microenvironment and microbiota could have long-lasting effects on dry eye conditions after COVID-19 infection. Some studies have shown that the use of probiotics and prebiotics can be effective in managing dry eye disease [29,30]. Furthermore, studies have suggested that the COVID-19 virus can alter the microbiota in the gut [31,32], which leads us to speculate that such alteration may also impact the microbiota in the eyes, causing inflammation and dysfunction of the meibomian glands. Therefore, it is important to further investigate the potential connection between ocular microbiota and their role in the development and management of dry eye disease after COVID-19 infection.

Many clinical studies have investigated the effects of the COVID-19 pandemic on dry eye diseases. Most of these studies focus on the impact of the pandemic on the general population, such as college students [33], nurses [34], healthcare professionals [35], or study students learning online for long distances [36]. The research methods primarily focus on questionnaire surveys. These studies mostly indicate that dry eye has worsened during the COVID-19 pandemic. However, there are fewer studies focusing on post-COVID patients, such as Turkey’s study [37], which found a shorter tear break-up time in post-COVID-19 patients; the UK’s study [38], which found sore eyes in COVID-19 patients; and China’s cross-sectional study [39], which found a higher incidence of DED symptoms in hospitalized patients with COVID-19. The innovation of our study is that we compare the dry eye parameters of the same patient before and after COVID-19, which further explains the impact of the coronavirus on dry eye.

This study has limitations that must be acknowledged. First, in order to determine whether eye-related symptoms persist in the long term, a larger sample size and multicenter, longitudinal study are needed. Second, since the time between the two tests exceeded one year, the influence of age on dry eye might not have been fully controlled. Although patients did not utilize any instruments for dry eye treatment, they frequently resorted to eye drops for relief. However, the lack of standardization in the brand of eye drops used by patients might have introduced some variation in the results [40]. Third, some of the patients were showing evidence of COVID-19 through rapid antigen testing, which made it difficult for us to obtain their viral load during their period of infection. Meanwhile, the vaccines might cause symptoms similar to infection [41], and the impact of vaccines on dry eyes requires further research.

In conclusion, our preliminary results found that after COVID-19 infection, patients with pre-existing dry eye disease experienced greater loss of meibomian glands, worsened conjunctival papillae, shorter tear break-up time, and particularly poorer lipid layer thickness, indicating a heightened severity of their existing dry eye condition. These finding highlight the need to raise awareness about the potential long-term effects of COVID-19 on dry eye, particularly among individuals who already suffer from the condition.

## Figures and Tables

**Table 1:** Demographic Characteristics in Post-COVID-19 dry eye patients and Non-COVID-19 dry eye patients.

	Post-COVID-19	Non-COVID-19	*p*-Value
Patient numbers	22	22	
Eye numbers	44	44	
Age (years)	38.36 ± 14.99	42.32 ± 17.52	0.372
Female: Male	18:4	15:7	0.296
Intervals times (months)			
1st Test to 2nd test	16.92 ± 5.40	12.94 ± 4.11	0.113
Infection to 2nd test	4.61 ± 2.39	NA	NA
COVID-vaccine (N%) ^#^			0.001
0 doses	15 (68.18%)	0 (0.00%)	
2 doses	3 (13.64%)	1 (4.55%)	
3 doses	3 (13.64%)	21 (95.45%)	
4 doses	1 (4.55%)	0 (0.00%)	

# Fisher’s exact test.

**Table 2:** Changes of the dry eye parameters in post-COVID-19 and non-COVID-19 dry eye patients.

	Post-COVID-19			Non-COVID-19		
	Before	After	*p*-Value ^#^	Baseline	Follow-Up	*p*-Value ^#^
Eye numbers	44	44		44	44	
Visual acuity (Log MAR)	−0.01 ± 0.14	0.02 ± 0.15	0.236	−0.02 ± 0.12	0.01 ± 0.15	0.130
OSDI	18.64 ± 18.15	14.68 ±12.99	0.133	20.68 ± 18.30	25.50 ± 19.32	0.063
NIKBUT-first(s)	9.74 ± 4.65	9.15 ± 5.75	0.612	9.97 ± 5.53	11.49 ± 6.22	0.133
NIKBUT-average(s)	14.26 ± 4.35	12.96 ± 4.82	0.164	14.30 ± 5.32	15.99 ± 4.79	0.059
Aqueous Parameters						
Schirmer’s Test (mm)	13.30 ± 9.21	14.48 ± 10.62	0.473	12.09 ± 10.32	13.02 ± 9.95	0.438
Tear Meniscus Height (mm)	0.21 ± 0.07	0.22 ± 0.07	0.252	0.28 ± 0.18	0.30 ± 0.19	0.193
Lipid Parameters						
LLT-average(nm)	63.00 ± 22.40	52.86 ± 18.00	<0.001	69.25 ± 23.87	74.41 ± 22.46	0.090
LLT-max(nm)	78.48 ± 20.55	67.89 ± 20.81	<0.001	80.73 ± 18.20	85.84 ± 18.41	0.077
LLT-min(nm)	46.41 ± 22.20	42.25 ± 15.06	0.140	57.82 ± 22.42	59.14 ± 22.51	0.718
Meibosocre upper eyelid (0–3)	1.55 ± 0.73	1.75 ± 0.84	0.011	1.39 ± 0.84	1.50 ± 0.73	0.133
Meibosocre lower eyelid (0–3)	1.30 ± 0.51	1.43 ± 0.73	0.001	1.23 ± 0.74	1.34 ± 0.61	0.133
Conjunctiva						
Papillae (0–3)	0.16 ± 0.37	0.61 ± 0.69	0.001	0.14 ± 0.41	0.30 ± 0.46	0.070
Follicle (0–3)	0.07 ± 0.33	0.27 ± 0.69	0.071	0.11 ± 0.39	0.16 ± 0.48	0.643

# Paired *t*-test. OSDI, Ocular Surface Disease Index; NIKBUT, Non-invasive Keratography break-up time; LLT, Lipid Layer Thickness.

**Table 3:** Comparison of the dry eye parameters difference in post-COVID-19 and non-COVID-19 dry eye patients.

	Post-COVID-19	Non-COVID-19	
	Difference	Difference	*p*-Value ^#^
Eye numbers	44	44	
Visual acuity (Log MAR)	0.03 ± 0.12	0.04 ± 0.15	0.734
OSDI	−0.86 ± 17.55	4.82 ± 16.71	0.124
NIKBUT-first(s)	−0.59 ± 7.67	1.52 ± 6.57	0.170
NIKBUT-average(s)	−1.30 ± 6.11	1.69 ± 5.78	0.020
Aqueous Parameters			
Schirmer’s Test (mm)	1.18 ± 10.83	0.93 ± 7.89	0.902
Tear Meniscus Height (mm)	0.02 ± 0.09	0.03 ± 0.14	0.619
Lipid Parameters			
LLT-average(nm)	−10.14 ± 16.29	5.16 ± 19.73	<0.001
LLT-max(nm)	−10.59 ± 15.48	5.11 ± 18.72	<0.001
LLT-min(nm)	−4.16 ± 18.36	1.32 ± 24.03	0.233
Meibosocre upper eyelid (0–3)	0.20 ± 0.51	0.11 ± 0.49	0.397
Meibosocre lower eyelid (0–3)	0.14 ± 0.59	0.11 ± 0.49	0.846
Conjunctiva			
Papillae (0–3)	0.45 ± 0.82	0.20 ± 0.59	0.105
Follicle (0–3)	0.20 ± 0.73	0.05 ± 0.65	0.283

# Student *t*-test. OSDI, Ocular Surface Disease Index; NIKBUT, Non-invasive Keratography break-up time; LLT, Lipid Layer Thickness.

**Table 4:** Association of COVID-19 infection and dry eye parameters difference in dry eye patients.

	Univariate Model			Multivariate Model		
	β	95%CI	*p*-Value	β	95%CI	*p*-Value
Visual acuity (Log MAR)	−0.01	−0.07, 0.05	0.765	−0.02	−0.08, 0.05	0.605
OSDI	−5.68	−15.69,4.33	0.266	−4.80	14.69, 5.09	0.342
NIKBUT-first(s)	−2.11	−5.21, 1.00	0.184	−1.98	−4.89, 0.92	0.181
NIKBUT-average(s)	−3.00	−5.78, −0.21	0.035	−2.98	−5.82, −0.15	0.039
Aqueous Parameters						
Schirmer’s Test (mm)	0.25	−3.94, 4.44	0.907	0.74	−3.31, 4.79	0.720
Tear Meniscus Height (mm)	−0.01	−0.07, 0.05	0.686	0.00	−0.06, 0.06	0.933
Lipid Parameters						
LLT-average (nm)	−15.30	−24.72, −5.87	0.002	−14.12	−22.66, −5.59	0.001
LLT-max (nm)	−15.70	−23.89, −7.52	<0.001	−15.65	−23.09, −8.20	<0.001
LLT-min (nm)	−5.48	−16.38, 5.42	0.325	−3.74	−14.39, 6.91	0.491
Meibosocre upper eyelid (0–3)	0.09	−0.17, 0.36	0.500	0.08	−0.19, 0.35	0.554
Meibosocre lower eyelid (0–3)	0.02	−0.25, 0.29	0.868	0.07	−0.18, 0.33	0.574
Conjunctiva						
Papillae (0–3)	0.25	−0.14, 0.64	0.210	0.22	−0.16, 0.60	0.257
Follicle (0–3)	0.16	−0.23, 0.55	0.425	0.18	−0.22, 0.58	0.370

Multivariable model adjusted for: Age, Sex; OSDI, Ocular Surface Disease Index; NIKBUT, Non-invasive Keratography break-up time; LLT, Lipid Layer Thickness.

## Data Availability

Not applicable.

## References

[1] Farrand K.F., Fridman M., Stillman I., Schaumberg D.A. (2017). Prevalence of Diagnosed Dry Eye Disease in the United States Among Adults Aged 18 Years and Older. Am. J. Ophthalmol..

[2] Markoulli M., Kolanu S. (2017). Contact lens wear and dry eyes: Challenges and solutions. Clin. Optom..

[3] Lin F., Cai Y., Fei X., Wang Y., Zhou M., Liu Y. (2022). Prevalence of dry eye disease among Chinese high school students during the COVID-19 outbreak. BMC Ophthalmol..

[4] Wolffsohn J.S., Arita R., Chalmers R., Djalilian A., Dogru M., Dumbleton K., Gupta P.K., Karpecki P., Lazreg S., Pult H. (2017). TFOS DEWS II Diagnostic Methodology report. Ocul. Surf..

[5] Tsubota K., Yokoi N., Watanabe H., Dogru M., Kojima T., Yamada M., Kinoshita S., Kim H.-M., Tchah H.-W., Hyon J.Y. (2020). A New Perspective on Dry Eye Classification: Proposal by the Asia Dry Eye Society. Eye Contact Lens.

[6] Uchino M., Nishiwaki Y., Michikawa T., Shirakawa K., Kuwahara E., Yamada M., Dogru M., Schaumberg D.A., Kawakita T., Takebayashi T. (2011). Prevalence and risk factors of dry eye disease in Japan: Koumi study. Ophthalmology.

[7] Takahashi Y., Vaidya A., Kakizaki H. (2022). Changes in Dry Eye Status after Steroid Pulse and Orbital Radiation Therapies in Active Thyroid Eye Disease. J. Clin. Med..

[8] Gandolfo S., Ciccia F. (2022). JAK/STAT Pathway Targeting in Primary Sjögren Syndrome. Rheumatol. Immunol. Res..

[9] Wan K.H., Lui G.C.Y., Poon K.C.F., Ng S.S.S., Young A.L., Hui D.S.C., Tham C.C.Y., Chan P.K.S., Pang C.P., Chong K.K.L. (2022). Ocular surface disturbance in patients after acute COVID-19. Clin. Exp. Ophthalmol..

[10] Yuki K., Fujiogi M., Koutsogiannaki S. (2020). COVID-19 pathophysiology: A review. Clin. Immunol..

[11] Ciotti M., Ciccozzi M., Terrinoni A., Jiang W.-C., Wang C.-B., Bernardini S. (2020). The COVID-19 pandemic. Crit. Rev. Clin. Lab. Sci..

[12] Kumar S., Thambiraja T.S., Karuppanan K., Subramaniam G. (2022). Omicron and Delta variant of SARS-CoV-2, A comparative computational study of spike protein. J. Med. Virol..

[13] Suryawanshi R.K., Chen I.P., Ma T., Syed A.M., Brazer N., Saldhi P., Simoneau C.R., Ciling A., Khalid M.M., Sreekumar B. (2022). Limited cross-variant immunity from SARS-CoV-2 Omicron without vaccination. Nature.

[14] Al-Aly Z., Bowe B., Xie Y. (2022). Long COVID after breakthrough SARS-CoV-2 infection. Nat. Med..

[15] Crook H., Raza S., Nowell J., Young M., Edison P. (2021). Long covid-mechanisms, risk factors, and management. BMJ.

[16] Desai A.D., Lavelle M., Boursiquot B.C., Wan E.Y. (2022). Long-term complications of COVID-19. Am. J. Physiol. Cell Physiol..

[17] Schiffman R.M., Christianson M.D., Jacobsen G., Hirsch J.D., Reis B.L. (2000). Reliability and validity of the Ocular Surface Disease Index. Arch. Ophthalmol..

[18] Li N., Deng X.G., He M.F. (2012). Comparison of the Schirmer I test with and without topical anesthesia for diagnosing dry eye. Int. J. Ophthalmol..

[19] Fukushima A., Ohashi Y., Ebihara N., Uchio E., Okamoto S., Kumagai N., Shoji J., Takamura E., Nakagawa Y., Namba K. (2014). Therapeutic effects of 0.1% tacrolimus eye drops for refractory allergic ocular diseases with proliferative lesion or corneal involvement. Br. J. Ophthalmol..

[20] Finis D., Pischel N., Schrader S., Geerling G. (2013). Evaluation of lipid layer thickness measurement of the tear film as a diagnostic tool for Meibomian gland dysfunction. Cornea.

[21] Arita R., Itoh K., Inoue K., Amano S. (2008). Noncontact infrared meibography to document age-related changes of the meibomian glands in a normal population. Ophthalmology.

[22] Fuller D.G., Potts K., Kim J. (2013). Noninvasive tear breakup times and ocular surface disease. Optom. Vis. Sci. Off. Publ. Am. Acad. Optom..

[23] Partridge L.J., Urwin L., Nicklin M.J.H., James D.C., Green L.R., Monk P.N. (2021). ACE2-Independent Interaction of SARS-CoV-2 Spike Protein with Human Epithelial Cells Is Inhibited by Unfractionated Heparin. Cells.

[24] Raghuvamsi P.V., Tulsian N.K., Samsudin F., Qian X., Purushotorman K., Yue G., Kozma M.M., Hwa W.Y., Lescar J., Bond P.J. (2021). SARS-CoV-2 S protein:ACE2 interaction reveals novel allosteric targets. eLife.

[25] Hamming I., Timens W., Bulthuis M.L.C., Lely A.T., Navis G.J., van Goor H. (2004). Tissue distribution of ACE2 protein, the functional receptor for SARS coronavirus. A first step in understanding SARS pathogenesis. J. Pathol..

[26] Shahraki T., Hassanpour K., Arabi A., Ansari I., Sadoughi M.M. (2021). Corona virus disease 2019-associated Stevens-Johnson syndrome: A case report. BMC Ophthalmol..

[27] Kapelushnik N., Benyosef S., Skaat A., Abdelkader A., Landau Prat D., Blum-Meirovitch S., Leshno A. (2022). The Effect of Face Masks during COVID-19 Pandemic on Ocular Surface Temperature-A Clinical Thermographic Analysis. Diagnostics.

[28] Mohammad Alrawashdeh H., Al Zubi K., Abdulmannan D.M., Al-Habahbeh O., Abu-Ismail L. (2021). Conjunctivitis as the only sign and symptom of COVID-19, A case report and review of literature. Qatar Med. J..

[29] Tavakoli A., Markoulli M., Papas E., Flanagan J. (2022). The Impact of Probiotics and Prebiotics on Dry Eye Disease Signs and Symptoms. J. Clin. Med..

[30] Andersson J., Vogt J.K., Dalgaard M.D., Pedersen O., Holmgaard K., Heegaard S. (2021). Ocular surface microbiota in patients with aqueous tear-deficient dry eye. Ocul. Surf..

[31] Mak J.W.Y., Chan F.K.L., Ng S.C. (2020). Probiotics and COVID-19, one size does not fit all. Lancet Gastroenterol. Hepatol..

[32] Yeoh Y.K., Zuo T., Lui G.C.-Y., Zhang F., Liu Q., Li A.Y., Chung A.C., Cheung C.P., Tso E.Y., Fung K.S. (2021). Gut microbiota composition reflects disease severity and dysfunctional immune responses in patients with COVID-19. Gut.

[33] Abdulmannan D.M., Naser A.Y., Ibrahim O.K., Mahmood A.S., Alkrad J.A., Sweiss K., Alrawashdeh H.M., Kautsar A.P. (2022). Visual health and prevalence of dry eye syndrome among university students in Iraq and Jordan. BMC Ophthalmol..

[34] Allayed R., Ayed A., Fashafsheh I. (2022). Prevalence and Risk Factors Associated with Symptomatic Dry Eye in Nurses in Palestine During the COVID-19 Pandemic. SAGE Open Nurs..

[35] Tangmonkongvoragul C., Chokesuwattanaskul S., Khankaeo C., Punyasevee R., Nakkara L., Moolsan S., Unruan O. (2022). Prevalence of symptomatic dry eye disease with associated risk factors among medical students at Chiang Mai University due to increased screen time and stress during COVID-19 pandemic. PloS ONE.

[36] Uzun S.L., Topcu H. (2022). The relationship of distance learning with ocular surface disorders in students in the COVID-19 pandemic. Int. Ophthalmol..

[37] Acet Y., Çil B., Kabak M., Vural E. (2022). Instability of Tear Film after Novel Coronavirus Disease: A Noninvasive and No Contact Method by a Scheimpflug-Placido Disc Topographer. Klin. Mon. Fur Augenheilkd..

[38] Pardhan S., Islam M.S., López-Sánchez G.F., Upadhyaya T., Sapkota R.P. (2021). Self-isolation negatively impacts self-management of diabetes during the coronavirus (COVID-19) pandemic. Diabetol. Metab. Syndr..

[39] Wang Y., Yang S., Zhang Y., Zhang X., Jiang Y., Wang X., Zheng P., Chen Y. (2021). Symptoms of Dry Eye Disease in Hospitalized Patients with Coronavirus Disease 2019 (COVID-19). J. Ophthalmol..

[40] Bayer I.S. (2022). Recent Advances in Mucoadhesive Interface Materials, Mucoadhesion Characterization, and Technologies. Adv. Mater. Interfaces.

[41] Inanc N., Kostov B., Priori R., Flores-Chavez A., Carubbi F., Szántó A., Valim V., Bootsma H., Praprotnik S., Trevisani V.F. (2022). Safety and efficacy of SARS-CoV-2 vaccination in 1237 patients with primary Sjögren syndrome. Clin. Exp. Rheumatol..

